# Understanding factors that influence the use of risk scoring instruments in the management of patients with unstable angina or non-ST-elevation myocardial infarction in the Netherlands: a qualitative study of health care practitioners’ perceptions

**DOI:** 10.1186/1472-6963-14-418

**Published:** 2014-09-22

**Authors:** Josien Engel, Marie-Julie Heeren, Ineke van der Wulp, Martine C de Bruijne, Cordula Wagner

**Affiliations:** EMGO Institute for Health and Care Research, Department of Public and Occupational Health, VU University Medical Center, Van der Boechorststraat 7, 1081 BT Amsterdam, The Netherlands; NIVEL, Netherlands Institute for Health Services Research, Utrecht, The Netherlands

**Keywords:** Risk assessment, Acute coronary syndromes, Guideline adherence, Implementation

## Abstract

**Background:**

Cardiac risk scores estimate a patient’s risk of future cardiac events or death. They are developed to inform treatment decisions of patients diagnosed with unstable angina or non-ST-elevation myocardial infarction. Despite recommending their use in guidelines and evidence of their prognostic value, they seem underused in practice. The purpose of the study was to gain insight in the motivation for implementing cardiac risk scores, and perceptions of health care practitioners towards the use of these instruments in clinical practice.

**Methods:**

This qualitative study involved semi-structured interviews with 31 health care practitioners at 11 hospitals throughout the Netherlands. Participants were approached through purposive sampling to represent a broad range of participant- and hospital characteristics, and included cardiologists, medical residents, medical interns, nurse practitioners and an emergency physician. The Pettigrew and Whipp Framework for strategic change was used as a theoretical basis. Data were initially analysed through open coding to avoid forcing data into categories predetermined by the framework.

**Results:**

Cardiac risk score use was dependent on several factors, including IT support, clinical relevance for daily practice, rotation of staff and workload. Both intrinsic and extrinsic drivers for implementation were identified. Reminders, feedback and IT solutions were strategies used to improve and sustain the use of these instruments. The scores were seen as valuable support systems in improving uniformity in treatment practices, educating interns, conducting research and quantifying a practitioner’s own risk assessment. However, health care practitioners varied in their perceptions regarding the influence of cardiac risk scores on treatment decisions.

**Conclusions:**

Health care practitioners disagree on the value of cardiac risk scores for clinical practice. Practitioners driven by intrinsic motivations predominantly experienced benefits in policy-making, education and research. Practitioners who were forced to use cardiac risk scores were less likely to take into account the risk score in their treatment decisions. The results of this study can be used to develop strategies that stimulate or sustain cardiac risk score use in practice, while taking into account barriers that affect cardiac risk score use, and possibly reduce practice variation in the management of unstable angina and non-ST-elevation myocardial infarction patients.

**Electronic supplementary material:**

The online version of this article (doi:10.1186/1472-6963-14-418) contains supplementary material, which is available to authorized users.

## Background

Cardiovascular diseases, including unstable angina (UA) and non-ST-elevation myocardial infarction (NSTEMI), are among the main causes of death of people across the world [1, 2]. International guidelines for the management of UA and NSTEMI [3–5] recommend to treat patients on the basis of their risk for adverse cardiac events such as re-infarction or death. High risk patients can be successfully treated with invasive procedures such as Percutaneous Coronary Intervention (PCI) or Coronary Artery Bypass Grafting (CABG). To accomplish this and to guide physicians in tailored therapeutic decision-making, several cardiac risk stratification scores have been developed [3, 5], i.e. the GRACE- [6, 7], TIMI- [8], PURSUIT- [9], FRISC- [10], and HEART [11] scores. Cardiac risk scores comprise of clinical factors associated with adverse cardiac outcomes [12]. The validity of these instruments in terms of their ability to predict the patient’s risk of re-infarction or death during hospitalization or after discharge was reported to be good [10, 11, 13–15]. Previous studies indicate that risk assessment based on physician’s experience was inferior compared to risk assessment by using validated risk scores [14, 16]. However, despite guideline recommendations and their prognostic value, these instruments are not widely adopted in clinical practice [17]. Practitioner related barriers e.g. knowledge, attitude, behavior, and external barriers related to the guideline, patient or organization, all affect guideline adherence by physicians [18]. Several studies reported low guideline adherence among physicians when managing UA/NSTEMI patients, resulting in a treatment risk paradox i.e. patients with a low risk of re-infarction or death were more likely to receive invasive treatment strategies (e.g. angiography and/or revascularization) compared to high risk patients [19–26]. Therefore, a gap between evidence-based care and routine clinical practice may exist which could affect patient outcomes negatively [21, 27–29]. To improve guideline adherence, quality improvement programs have been initiated in several countries [30–33]. Recently in the Netherlands, such a program was introduced in which, among other things, the use of cardiac risk scores was evaluated [34]. However, to our knowledge, it is unknown to what degree healthcare professionals’ perceptions regarding the value of cardiac risk scores in therapeutic decision making may affect the use of these scores in clinical practice. There is also little understanding of factors that facilitate or hinder health care practitioners in their attempts to implement these risk scores in practice. Therefore the objectives of the study are to gain insight in the motivation for implementing cardiac risk scores, and perceptions of health care practitioners towards the use of these instruments in clinical practice.

## Methods

### Study design and setting

A qualitative study involving semi-structured interviews was conducted. Professionals employed at cardiology departments of hospitals that previously participated in the evaluation of a Dutch quality improvement program (n = 13), were approached for participation in this study. This program aimed to optimize care for patients diagnosed with acute coronary syndromes, including UA and NSTEMI, and is based on the recommendations of the European Society of Cardiology guidelines. The hospital sample was verified to be representative for the Dutch population of hospitals, with regard to type of hospital, e.g. teaching versus non-teaching, and the availability of specific cardiac facilities, e.g. PCI or CABG.

### Study participants

In each hospital, the cardiologist who was a contact person for the Dutch quality improvement program was approached for participation in the present study. They were selected because they were involved in implementing a cardiac risk score in their institution. After each interview they were asked to recruit or provide contact details of a colleague within their department. They were subsequently approached directly by the researcher (JE) during site visits or by email. Participants were eligible if they were a) currently employed in one of the participating hospitals, b) directly involved in the treatment of UA/NSTEMI patients, i.e. physicians or nurses, c) regardless of their attitude/opinion were experienced in using cardiac risk scores and/or d) involved in the implementation of a cardiac risk score. By means of purposive sampling, the selection of participants ensured diversity on the type of profession, their level of work experience and the type of hospital they worked in.

### Development of interview protocol

The interview protocol was structured according to the three dimensions of the Pettigrew and Whipp framework i.e. context, process and content [35] and by reviewing literature about implementation strategies and corresponding barriers and facilitators (Additional file 1) [36–39]. For the present study, the three dimensions of the framework were interpreted as following: *context*; what are motivations behind the use of cardiac risk scores?, *process*; what strategies are applied to enable, enhance and/or sustain cardiac risk score use and which factors influence this process?, *content*; what are opinions of health care practitioners towards the value of cardiac risk scores for clinical practice and which effects did they perceive? The interview protocol was pilot-tested with an emergency physician who was involved in the implementation of a cardiac risk score, but was not part of the current research sample. In addition, the adequacy and functionality of the revised interview protocol was discussed within the research team until consensus was reached.

### Data collection

Semi-structured interviews were conducted between September 2012 and May 2013. Data were collected on site or at the participant’s home. Prior to the interview, participants received an information letter explaining details about the study. All interviews were audio-recorded and transcribed at verbatim unless participants objected. In the latter case, hand written notes were made and a detailed transcription was sent back to the participant for verification (n = 1). Interviews were conducted by one member of the research team (JE) who was trained in qualitative interviewing.

### Qualitative data analysis

The transcribed interviews were initially analyzed using open coding to avoid forcing data into the predetermined categories i.e. context, content and process. The first five transcribed interviews were coded by two researchers independently, to form an initial code list and to enhance reliability of the analyses process (JE, MJH). Differences between the coding’s of the researchers were resolved in consensus meetings. During the analyses of subsequent interviews, the initial code list was further refined by adding new codes or reconstructing existing codes. The definitions of the final code set and the hierarchy of the code structure were reviewed for logic. The final version of the code structure was applied on all transcribed interviews (JE). To ensure concordance in codings, 50% of the transcriptions were coded independently by a second researcher (MJH). Relevant differences in applying the final code structure were discussed and resolved. All transcriptions were reviewed with the revised final code structure by one researcher (JE). To determine if the code structure was sufficient and to ensure no new information occurred (i.e. saturation), three additional interviews were subsequently conducted, transcribed and analyzed [40]. All data were analyzed in Atlas.ti V.5.7 (ATLAS.ti Scientific Software Development Company, GmbH, Berlin, Germany).

#### Validation and reliability

Several techniques were used to enable a systematic and transparent process of data collection and analyses. First, after each interview field notes were made which included factual data regarding the interview-setting, observations during the interview, and reflective information regarding thoughts and concerns. They were used to interpret the data more carefully. Second, the interview protocol was consistently used and critically reviewed after each interview. Third, two researchers coded the transcribed interviews independently in ATLAS.ti to manage the coding process. Finally, consensus meetings were held to discuss and reconcile differences in coding of the data. Analytical decisions made in the consensus meetings were documented.

### Ethics

Ethics approval was obtained from the medical ethical committee of the VU University Medical Center Amsterdam. Written informed consent for participation and audio-taping of the interview was obtained from all respondents. Confidentiality was assured by removing traceable information from transcripts relating to participating hospital sites or individuals. Data were stored on a protected network server at the research institute, only accessible to the research team.

## Results

**Figure 1:**
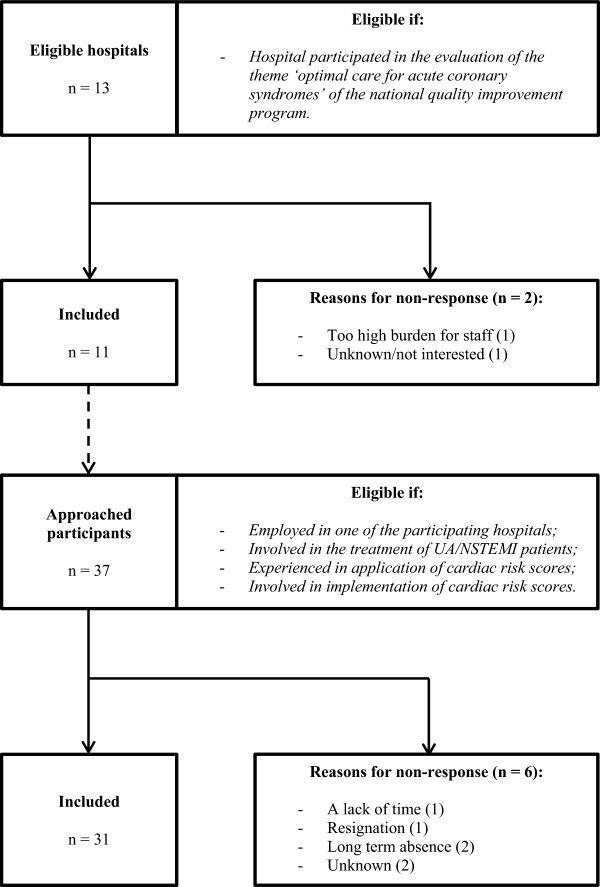
**Flow diagram of hospital and participant selection.**

Interviews were conducted at 11 hospitals. Two teaching hospitals with invasive treatment facilities on site refused to participate. One hospital considered interviews too much of a burden for staff, the other hospital did not provide a reason for refusal. In total 37 health care professionals were approached, of which 16 cardiologists, seven medical residents, four medical interns (including one research fellow), three nurse specialists and one emergency physician, were interviewed (Table 1). They were familiar with either the GRACE-, TIMI-, FRISC- or HEART risk score at their institution. Six participants could not be interviewed, due to among other a lack of time, resignation or long term absence (Figure 1). The average length of an interview was approximately 30 minutes, however, substantial variations in length occurred*.* The analyses resulted in nine main categories fitted in the dimensions of the Pettigrew and Whipp Framework (Table 2). These are elaborated below and illustrated by representing quotations (Additional file 2: Table S3).

**Table 1:** **Hospital and participant characteristics**

Hospital characteristics	No. (%) of hospitals ^a^
	(n = 11)
Type of hospital	
Teaching	7 (63.6)
Non-teaching	4 (36.4)
Facilities	
PCI	2 (18.2)
PCI and CABG	3 (27.3)
No revascularization facilities	6 (54.5)
**Participant characteristics**	**No. (%) of participants** ^**a**^
	**(n = 31)**
Gender	
Male	21 (67.7)
Age (years)	
Mean (SD)/Range	38.9 (9.4)/26-61
Type and years in profession^b^	
Cardiologists	16 (51.6)
<5	5 (31.25)
5-10	5 (31.25)
>10	6 (37.5)
Medical resident	7 (22.6)
<5	6 (85.7)
5-10	1 (14.3)
> 10	n.a.
Medical intern	4 (12.9)
< 5	3 (75)
5-10	1 (25)
> 10	n.a.
Nurse specialist	3 (9.7)
< 5	1 (33.3)
5-10	2 (66.7)
> 10	n.a.
Emergency physician	1 (3.2)
< 5	n.a.
5-10	1 (100)
> 10	n.a.
Length of interview (minutes)	
Median (IQR)	28.2 (25.6)
< 15	9 (29)
15-30	8 (25.8)
30-45	10 (32.3)
45-60	3 (9.7)
>60	1 (3.2)

^**a**^In no. (%), unless stated otherwise; ^**b**^Years in current profession/position. Abbreviations: PCI, percutaneous coronary intervention; CABG, coronary artery bypass grafting; n.a., not applicable.

**Table 2:** **Themes, categories and concepts**

PGF dimensions ^ab^	Category	Description	Concepts
WHY context	Intrinsic motivations	Personal beliefs of health care practitioners that leads to the implementation	Uniformity problem
I. Stimuli for implementing cardiac risk scores			Educational support
			Research purposes
	Extrinsic motivations	Environmental and organizational pressure that leads to the implementation	(Inter) national guideline recommendations
			Governmental pressure and regulatory demands: quality improvement program, recommendations of Dutch association of cardiology, audits of health care inspectorate
			Pressure hospital board
			Assessments by health care insurance companies
HOW process	Implementation strategies	Interventions used to enhance or support the implementation process	Support and commitment staff
II. Process of implementing cardiac risk scores			Clinical reminders: posters (passive), written and oral reminders (active)
			Data feedback
			Education: practical and theoretical
			Development project plan
			Appointment working committee
	Facilitators and barriers	Influential factors enhancing or hindering the implementation process	*Facilitating factors*
			Innovation level: clinical relevance
			Practitioner level: commitment staff
			Organization level: management support, IT support
			*Barriers*
			Innovation level: administrative burden, complexity of underlying algorithm of risk score, loss of time
			Practitioner level: level of work experience, familiarization with new practices, lack of knowledge, lack of relevance
			Organization level: frequent staff rotation, high work load, lack of time, lack of management priority, lack of resources, fast update of guidelines, unexpected circumstances
	Sustainability	Interventions undertaken to sustain change in practices	Redesigning systems: integration of risk score(s) in existing electronic hospital systems, protocols or clinical pathways
			Audit and feedback
			Appointment of champions
WHAT content	Choice of risk score	Motivation for implementing cardiac risk score and its use in practice	Choice of risk score based on: purpose, availability relevant parameters, complexity, validity and available scientific evidence, recommendations of clinical guidelines, accordance own practices
III. Perceptions of health care practitioners			
			Use in practice: type of risk score (GRACE, TIMI, FRISC or HEART), intended users (interns, residents, less often cardiologist, nurse specialists), target group (patients with chest pain, unstable angina, non-ST-elevation myocardial infarction or acute coronary syndrome), location (emergency department, chest pain unit, coronary care unit)
	Unintended benefits and risks	Implementation effects in terms of benefits and risks for quality and safety of care	Expected benefits: improved uniformity, educational support, scientific benefits
			Unintended benefits: support system, enhanced patient safety
			Risks: regulatory medicine
	Impact on treatment policies	Impact on physician’s decision-making process in terms of admission and treatment policies	Treatment policy: no consequence, conservative treatments (pharmacological), invasive treatments (cardiac catheterization or revascularization)
			Admission policy: admission protocol, patient allocation, patient flow
	Effects on process of care	Effectiveness of cardiac risk score implementation	Current practice and variation in practice

^**a**^Pettigrew & Whipp framework. ^**b**^The provided information cuts across more than one dimension.

### I Stimuli for implementing cardiac risk scores *(context)*

Two types of stimuli to implement cardiac risk scores were reported by participants: *intrinsic motivations* i.e. from within the department and *extrinsic motivations* i.e. external pressure. In most cases both factors were drivers for cardiology departments to implement a cardiac risk score instrument.

#### Intrinsic motivations

The need for a more uniform approach in admission and treatment practices for patients presenting with suspected UA or NSTEMI was the most commonly mentioned motivation for implementing a cardiac risk score. Also, educational purposes were a frequently mentioned motivation. It was expected that the use of cardiac risk scores created awareness among less experienced physicians in estimating patients’ risk of re-infarction or death. Finally, cardiac risk scores were considered of value for scientific research in which they were used to determine the characteristics of the patient population.

#### Extrinsic motivations

External pressures such as the incorporation of cardiac risk score use in European Society of Cardiology guidelines accelerated the implementation process in several hospitals. In addition, a national quality improvement program stimulated the use of these guidelines and, partly due to its obligatory character, all hospitals aimed to follow these recommendations. Some participants experienced additional pressure from their hospital board to comply with the requirements of the quality improvement program. Other less frequently mentioned pressures were recommendations of the Dutch Association of Cardiology, regulatory audits from the health care inspectorate, and performance assessments by health care insurance companies.

### II Process of implementing cardiac risk scores *(process)*

Participants mentioned three complementary categories, which determine the process of implementation: *implementation strategies*, *barriers and facilitators* and *sustainability*.

#### Implementation strategies

Support of senior staff was considered effective in enhancing the implementation and was accomplished by actively referring to cardiac risk scores e.g. during hand-off sessions. Written reminders to the entire team were applied to pay attention to non-compliance. Also, individuals were personally addressed by one of the senior staff members. Several hospitals used regular data feedback as a strategy to motivate colleagues. To build a consistent knowledge base among medical residents and interns, in all hospitals lectures and personal or written instructions were provided. Finally, other incidentally mentioned interventions, as part of the implementation strategy, were: developing a project plan, establish a working committee and the use of passive reminders, e.g. posters.

#### Facilitators and barriers

Respondents mentioned that resistance in applying cardiac risk scores was related to the absence of a clinical consequence or critics against the available scientific evidence for using these instruments. Stressing the clinical relevance and importance, especially by the senior staff, was therefore considered crucial in reducing resistance. Also the administrative burden and complexity of risk score calculations affected its use. In some hospitals this was solved by support from the hospital management board and information technology (IT) department by integrating the calculation and registration of cardiac risk scores in existing software platforms. However, for some risk scores the underlying algorithms were not directly accessible which delayed IT integration.

In explaining low compliance rates, several respondents mentioned that the value of a cardiac risk score in practice was dependent of the professional’s experience in cardiology. For example, medical interns were frequently mentioned as benefitting most from using a risk score in founding their treatment decisions in contrast to experienced cardiologists. Moreover, participants noticed that less experienced cardiologists were generally more familiar with clinical prediction models compared to the older. The latter group familiarized themselves more slowly with a cardiac risk scoring instrument. Another barrier in implementing cardiac risk scores was the frequent rotation of medical interns. Continuous education and reminders were necessary to support and sustain the use of cardiac risk scores. Also a high-workload and a lack of available time were frequently mentioned as hindering factors in the application of the risk score. Some cardiologists expressed that external pressures, such as audits, were necessary to be given priority and to receive support of the hospital management board. Other less frequently experienced barriers were a lack of available resources including finances and personnel, lack of relevance (e.g. absence of on-site revascularization options or number of employed cardiology residents), frequent updates of the guidelines and unexpected circumstances including the absence of key persons due to sick leave.

#### Sustainability

Although most hospitals were in the process of integrating cardiac risk scores in clinical practice, specific strategies were applied to maintain its use on the long term. IT solutions to incorporate cardiac risk scores in the hospital system, including triggers, links and mandatory fields, were helpful reminders. Hospitals without such facilities integrated the cardiac risk score in existing clinical pathways or protocols. Another strategy to maintain cardiac risk score use was periodic audit and feedback sessions. Finally, in some hospitals champions, e.g. a nurse specialist or research fellow, supported by a cardiologist monitored the implementation.

### III Perceptions of health care practitioners *(content)*

Perceptions of health care practitioners regarding cardiac risk scores and their use could be allocated in four categories: *choice of risk score*, *unintended and intended benefits and risks*, *impact on treatment policies,* and *effects on the process of care.*

#### Choice of risk score

Hospitals aimed to apply cardiac risk scores when patients presented at the emergency department, chest pain unit or the coronary care unit with a suspected or confirmed diagnosis of UA or NSTEMI. Aspects determining the choice for a specific cardiac risk scoring instrument were the purpose of the risk score, availability of the parameters necessary to determine patients’ risk, guideline recommendations and scientific evidence. Most hospitals implemented the GRACE risk score. However, applicability of the GRACE was limited due to its dependency on calculators and IT solutions. Some hospitals therefore implemented the TIMI, FRISC or HEART score. Hospitals choosing for the latter preferred a tool that was suitable for a broader category of patients i.e. patients presenting with chest pain to the emergency department.

#### Unintended and intended benefits and risks

Participants mentioned that implementing a cardiac risk score instrument improved uniformity in treating UA and NSTEMI patients. As a result, participants believed risk scores enhanced patient safety and efficient resource use. Moreover, cardiac risk score use led to a more rapid recognition of high risk patients and created awareness regarding the appropriate site of care. Among interns, cardiac risk scores provided a more clear understanding of the departments’ standards regarding the care for UA and NSTEMI patients and increased their awareness of the factors associated with a high risk of adverse cardiac events. Also, its use gave hospitals the opportunity to study illness severity among their population of patients. Participants indicated that the risk score instrument was used as an objective support system to quantify their risk assessment, to confirm their assumptions regarding a patient’s risk and/or to justify their chosen treatment plan. Possible risks associated with cardiac risk score use were related to overregulation of the process of care e.g. because participants indicated that mortality risk may be overestimated. Therefore, they believed that treatment policies should not be solely based on a risk score.

#### Impact on treatment policies

Participants reported variation in the degree cardiac risk scores affected the choice between the treatment options. Some participants continued to use conventional risk stratification and clinical experience solely. Others used the risk score as a guide in their decision making, combined with conventional risk stratifiers. In the latter case, cardiac risk scores were mainly used to identify high risk patients who would benefit most from aggressive and timely treatment. In patients with high age, severe heart failure, cognitive impairments and immobility, physicians often deviated from the guidelines as cardiac risk scores could not comprehend the full spectrum of UA or NSTEMI presentations. A few participants mentioned that the risk score also influenced their admission protocol and patient flow. Participants described adjustments in their admission protocols according to the calculated risk score outcome, for instance low risk patients were either sent home, treated at the outpatient department or admitted to the hospital. Cardiac risk scores were also used to guide patient admission to appropriate sites of care or to enhance the throughput of patients on the emergency department.

#### Effects on process of care

The implementation of cardiac risk scores resulted in most hospitals in a more uniform approach in supervising interns and in the assignment of (invasive) treatments, though this was disputed by a few participants. They questioned whether hospitals would continue to use cardiac risk scores in daily practice if the national quality improvement program stopped. Actually, a division was observed between hospital departments which implemented a risk score for registration purposes solely, and hospitals in which the guideline recommendations were strictly followed.

## Discussion

This study investigated perceptions of health care professionals concerning the implementation and use of cardiac risk scores in the management of patients diagnosed with UA or NSTEMI.

It appeared that the active involvement of staff members, and the presence of champions responsible for data feedback, sending clinical reminders, education of colleagues and promoting cardiac risk score use on their department were strategies used to implement cardiac risk scores. These were also found in previous studies regarding the evaluation of guideline implementation in cardiology [31, 41, 42], or guideline dissemination in general [37, 43]. In implementing cardiac risk scores, two crucial factors in sustaining their use were mentioned i.e. IT support arranged and prioritized by the hospital board and emphasizing the clinical relevance of the risk score. Apart from the frequent rotation of medical interns, similar barriers in guideline implementation have been reported previously [18, 44]. In most hospitals the frequent rotation of medical interns resulted in periodic knowledge deficits which hindered efforts to sustain cardiac risk score use. Previous research regarding underperformance of medical interns or residents identified, among other things, a lack of medical knowledge and poor decision making and clinical judgment skills as underlying problems of underperformance [45, 46]. This emphasizes the importance of constant education and feedback in sustaining cardiac risk score use in clinical practice. It is recommended that future quality improvement initiatives take the aforementioned barriers and strategies into account when aiming to improve cardiac risk score use in clinical practice. In addition, future updates of the ESC guidelines could emphasize effective strategies to facilitate cardiac risk score implementation. However, further research is needed to assess the impact of the suggested strategies on risk score adherence.

The results in this study further show that in clinical practice cardiac risk scores were often used as intended, though the impact of the resulting scores on treatment decisions varied and depended highly on the patient’s risk of adverse cardiac outcomes. This is in accordance with the European Society of Cardiology guidelines, which recommend to administer therapies tailored to a patient’s level of risk [5]. However, it has been reported previously that beliefs about practice and actual practice differ substantially [41]. It is therefore unknown to what degree cardiac risk scores affect clinical decision-making in relation to other information such as electrocardiogram findings or the presence of co-morbidities. This should be studied further. Apart from the risk score’s influence on treatment practices, the scoring instruments also functioned as objective support systems in quantifying, confirming and/or justifying physicians’ initial risk assessment. Additional benefits, included improved uniformity in treatment practices, educational support and scientific support. These benefits were in concordance with intrinsic motivations of participants prior to risk score implementation. In addition, practitioners who felt forced to use cardiac risk scores were less likely to take into account the cardiac risk score in their treatment decisions or saw a benefit of cardiac risk score use in their own practice, and continued to use conventional risk stratification and base decision making on clinical experience solely. It is therefore recommended for hospital management staff to emphasize and disperse information about these potential benefits of using risk scores throughout their organization.

### Limitations

In interpreting the results of this study, several limitations should be taken into account. First, to structure the contents of the interviews, the dimensions of the Pettigrew and Whipp framework were slightly deviated from the original framework. This resulted in a thorough analysis of practices in each hospital. Second, the length of interviews differed considerably between respondents that may have influenced the quality of the data. It appeared that knowledge regarding the implementation of cardiac risk scores differed substantially between participants. Also, some interviews were interrupted because of acute patient admissions. Of these, memo’s and transcripts were critically reviewed. Where deemed necessary, follow-up interviews were planned. Finally, participant checks to enhance external validity were not conducted (except in case the interview was not audio-taped), among other things, because of the likelihood that participants changed their views over time. The information that emerged from the interviews may therefore not be representative for all practitioners involved in the management of patients diagnosed with UA or NSTEMI, and may differ for hospitals not involved in the study. However, we presume these differences to be negligible due to the diversity in participant characteristics and because saturation was obtained. In addition, it was assumed that audio-taping of the interviews and transcribing verbatim contributed in great extent to the validity of the study results. Also, the use of risk scores is embedded in several international cardiac guidelines. In the Netherlands, it is strongly recommended to use the European Society of Cardiology guidelines in the management of UA and NSTEMI patients. The results of this study could therefore be of use for all practitioners applying these guidelines in the management of UA or NSTEMI patients as the context of care is comparable.

## Conclusions

Health care practitioners disagree on the importance of cardiac risk scores used to decide on the management of unstable angina or non-ST-elevation myocardial infarction patients. Practitioners predominantly experienced benefits in policy-making, education and research when intrinsic motivations were underlying the implementation of cardiac risk scores. In addition, practitioners who felt forced to use cardiac risk scores were less likely to take into account the cardiac risk score in their treatment decisions. The study results can be used to develop effective strategies that stimulate or sustain cardiac risk score use in future practice and reduce practice variation in the management of UA and NSTEMI patients. These strategies may be incorporated in future updates of the ESC guidelines, as currently these do not contain information on how to implement cardiac risk scores in clinical practice. However, several barriers that affect implementation and applicability in practice need to be taken into account.

## Electronic supplementary material

Additional file 1: **Key informant interview guide.** Key informant interview guide based on the WHY/HOW/WHAT dimensions of the Pettigrew and Whipp framework for strategic change and existing implementation literature. (PDF 11 KB)

Additional file 2: Table S3: Representative quotations. Illustrating quotations of interviewees. (PDF 31 KB)

## Abbreviations

UA: Unstable angina
NSTEMI: Non-ST-elevation myocardial infarction
PCI: Percutaneous coronary intervention
CABG: Coronary artery bypass grafting
IT: Information technology
ESC: European Society of Cardiology.
