# Supplementary material for: Understanding factors that influence the use of risk scoring instruments in the management of patients with unstable angina or non-ST-elevation myocardial infarction in the Netherlands: a qualitative study of health care practitioners’ perceptions
Source: BMC Health Serv Res. 2014 Sep 22;14:418. doi: 10.1186/1472-6963-14-418 (PMC4263206; doi:10.1186/1472-6963-14-418)
Supplement: Supplementary file 1 — Additional file 1: Key informant interview guide. Key informant interview guide based on the WHY/HOW/WHAT dimensions of the Pettigrew and Whipp framework for strategic change and existing implementation literature. (PDF 11 KB) [file 12913_2014_3525_MOESM1_ESM.pdf]

Appendix 1: Key informant interview guide based on the WHY/HOW/WHAT dimensions of the Pettigrew and Whipp framework for strategic change and existing implementation literature (variation in questions asked depended on participants role).

1. **WHY:** What was/were (the) specific motivation(s) for change: why did hospitals wish to implement a cardiac risk score?

    a. At the department level.

    "What was the main incentive to start or continue cardiac risk score implementation in your department?"

    b. In terms of the external context.

    "Has the implementation been guided by factors at the national or organizational level?"

2. **HOW**: What implementation efforts were undertaken to facilitate implementation or to sustain implementation regarding cardiac risk scores?

    a. Effective implementation strategies.

    "Which interventions were applied to implement a cardiac risk score?"

    "Which of these interventions enhanced the implementation process?"

    b. Perceived implementation-related facilitators and barriers.

    "What facilitated implementation activities in your department?"

    "What hindered implementation activities in your department?"

    "What could have been done differently?"

    c. Resource utilization and management support

    "Did you receive management support at the organizational level, if so in what way?"

    "Where necessary resources available for successful implementation?

    "Did intended users receive proper training regarding the use and purpose of cardiac risk score instruments?"

    d. Sustain change.

    "What activities have been taken place to ensure cardiac risk score use over time?"

    "Has the use of cardiac risk scores became part of the risk stratification process at your department?"

3. **WHAT**: What where the perceptions of health care providers regarding cardiac risk scores and what unintended and intended benefits or risks did they experience?

    a. In terms of prior expectations

    " What were expectations prior to implementation and in what extent did they came true?"

“What is, if so, the additional value of a cardiac risk score to the usual risk stratification process that already existed in your department?”

b. In terms of additional value for risk clinical practice

“What were the effects of introducing cardiac risk scores for your department?”

“What benefits do cardiac risk scores possible bring or brought for your department?”

“What disadvantages do cardiac risk scores possible bring or brought for your department?”

“Has the implementation of a cardiac risk score affected culture or habits in your department? If so, describe the shift?”

c. In terms of application in practice

“What was/were motivation(s) to choose a specific type of cardiac risk score?

“How is the score applied in practice (type of risk score, target group, intended users, location)?

“How do you perceive the user-friendliness of the instrument?”

“Can you describe current practices regarding cardiac risk score use?”

Source: Pettigrew AM, Whipp R. Managing Change for Competitive Success. Oxford: Blackwell Publishing; 1993.
