# Supplementary material for: Understanding factors that influence the use of risk scoring instruments in the management of patients with unstable angina or non-ST-elevation myocardial infarction in the Netherlands: a qualitative study of health care practitioners’ perceptions
Source: BMC Health Serv Res. 2014 Sep 22;14:418. doi: 10.1186/1472-6963-14-418 (PMC4263206; doi:10.1186/1472-6963-14-418)
Supplement: Supplementary file 2 — Additional file 2: Table S3: Representative quotations. Illustrating quotations of interviewees. (PDF 31 KB) [file 12913_2014_3525_MOESM2_ESM.pdf]

Table 3 Representative quotations

PGF dimension	Category	Representative quotes
I. Stimuli for implementing cardiac risk scores *(context)*	Intrinsic motivations	*In practice we ran into an uniformity problem regarding admission decisions and choice of drug therapy. We wanted to translate the structure that you have in your head as a physician, when making a risk assessment, to a score (…). This is often a feeling, while a score is a way to structure this, to justify (…). In the past it was not clear where choices were based on (…). This led to uncertainty and a lack of clarity among the medical interns working at the emergency department (Cardiologist, teaching hospital, PCI facilities).*
		…in particular to explain to interns, that this is a risk stratification model, which can be used to determine the risk of mortality and that it may have implications for your treatment. More as a tool for education I think, than that we often based (treatment) decisions on it in the past (cardiologist, teaching hospital, PCI facilities).
		Actually, it started as just registering risk factors for scientific purposes, not so much for practice purposes. We started with the TIMI early 2000 (…) with the idea to use it for research and to compare patient groups (cardiologist, teaching hospital, PCI facilities).
	Extrinsic motivations	*The latest guidelines, of last year, indicate that you should perform risk stratification. It is up to yourself to determine how you accomplish that. A risk score is most convenient. (…) It is possible that the quality improvement program was an extra stimuli. However, complying with the guidelines is part of your job, so… (cardiologist, teaching hospital).*
		It is, in particular, introduced because of the fact that it is an indicator of the quality improvement program. I honestly think that otherwise, in most clinics in the Netherlands, it would be without obligations. And that is no more (cardiologist, teaching hospital, PCI facilities).
		First, these sort of things (i.e. quality indicators) are requested from authorities e.g. health care insurances and health care inspectorate. Second the standardization of treatment, an unambiguous policy. Even among us (i.e. cardiology staff) (cardiologists, general hospital).
		The manager intensive care has told the cardiologists: 'these are the requirements of the quality improvement program, where you have to start working on' (emergency physician, general hospital).
II. Process of implementing cardiac risk scores *(process)*	Implementation strategies	*So, what was my role in it? I have presented the guidelines and the GRACE score to the staff, held a few presentations about it, discussed all the guidelines and then we decided (with fellow cardiologists) to implement the new guidelines in practice. (..) First you have to agree as a team that you are going to use it. Second, that you have to explain what the GRACE is, where it comes from and what the reason behind the implementation is (cardiologist, teaching hospital).*

Table 3 Representative quotations (continued)

PGF dimension	Category	Representative quotes
II. Process of implementing cardiac risk scores *(process)*	Implementation strategies	*Well, by using data feedback, every certain period, regarding how often it is filled out (the risk score instrument) and how often it isn't. Than you see that people get more and more aware of the fact that it is controlled: 'people are looking at our work'. Well, then you can discover a learning curve. People are becoming more aware and start filling it out (nurse specialist, teaching hospital, PCI facilities).*
	Facilitators and barriers	*That you really need it to make a treatment decision. I can imagine, if that is not the case, that at one point the (…) score will no longer be used. That there will be a re-lapse on conventional risk factors (cardiologist, teaching hospital).*
		(…) once again you must link it to a policy change. So you have to say in case of a low score we do this and in case of a high score we do that. As long as you don't do that, it has no point, except for registration. (…) It should be an incentive to implement something in which you can improve care. As long as you only implement it to register: waste of time (research fellow, teaching hospital, PCI facilities).
		(…) Look, some of the data should be automatically extracted with that electronic file of ours. So, basically, blood pressure, heart rate, age and renal function, can all be extracted without you having to think about it. And then, you make it (a) mandatory and (b) easy. Then you can do so much more with it (cardiologist, teaching hospital).
		If the bosses (staff) don't ask for it, then it's gone within two weeks. So, it must be useful for the patient, that is motivation number one. And if it is really useful, everybody will continue using it by himself of course. If it is a bit more questionable, you need someone to sit behind you rags and immediately point it out to you. Especially if it is the boss himself. If that is absent as well, than such a registration is doomed. Nothing will happen anymore (research fellow, teaching hospital, PCI facilities).
		There is a fast rotation of interns, which hinders the introduction and sustainability of an instrument. I continuously have to point out the use of the instruments, until this leads to saturation. Once the acquaintance is there, a new group of interns arrives. This makes it difficult. Also, there is a lack of knowledge among the interns: a lot of newcomers in a short period of time (cardiologists, teaching hospital, PCI facilities).
		High workload. And I must say that the interns fill it out very well. Maybe it is more a point of attention for the cardiologists. But I have no evidence for that (nurse specialist, general hospital).
		(…) There are people who really feel summoned to apply the HEART score, and others think 'for me this is not necessary'…..or 'I will do this at the nursing ward'. They don't understand the sooner you sustain a trajectory, it is just finished. That's what I notice. Young cardiologists are educated with safety management systems and criteria you have to pay attention to. More conservative specialists, who have been working here for a long time, but that counts for all specialism's, say: 'we do that for years, why should we adjust that?' (emergency physician, general hospital).

Table 3 Representative quotations (continued)

PGF dimension	Category	Representative quotes
II. Process of implementing cardiac risk scores *(process)*	Sustainability	*And I do have the idea that everybody tries to fill them in as best as possible. But look, it (risk score) is not integrated in the [name electronic patient file], which of course would be fantastic. If you admit someone with an acute coronary syndrome and then get such a standard fill out table. Then, I think, it will always be done well (medical intern, teaching hospital).*
		Namely nurse specialists are very suitable for that, they are good in reasoning from protocol and in mapping of these trajectories. They are trained to implement that both in the nursing echelon as in the medical. And in that manner nurse specialists are a valuable addition for our clinical operations (cardiologists, general hospital).
III. Perceptions of health care providers *(content)*	Choice of risk score	*That one (i.e. GRACE) is more extensively validated, more accurate, more well-known, plus it is recommended as first choice by the guidelines. It is more useful for the clinic, than the FRISC score I think. But he is slightly more complicated. (research fellow, teaching hospital, PCI facilities)*
		The considerations for risk stratification is, at this moment, that the TIMI score is a more simpler tool and especially because there is too little support from the IT department to support the GRACE. That actually means that it is more convenient for your normal workflow to choose the TIMI score. While we actually have seen that the GRACE score is more often used and also should be, within our guidelines, the recommended risk score (…) (cardiologist, general hospital).
		Well… it is (HEART score) well applicable in the group of patients that we get presented on the emergency department. While the GRACE and in particular the TIMI are much more focused on a selected group of patients who….yeah, a bit disrespectful put, you already know that you have to act acutely on. While it is, especially with the group of interns we have here, important to correctly select the right group of patients arriving at our emergency department (cardiologist, general hospital).
	Unintended and intended benefits and risks	*It's just easy, I find, in the work process if you can apply scores. If you work with young people, let me put it in this way, then protocols, guidelines and scores are easy for decision making. And I work here with young people (emergency physician, general hospital).*
		In their thinking- and learning process that pink form (i.e. risk score) works extremely well. Because, we ordered to fill it out, but what does it mean? They have to immerse oneself in it. They receive some explanation, but after that they have to apply it themselves. So for interns it is a very good learning tool (nurse specialist, general hospital).
		Yes, well another benefit is when you start doing research. Database research at yourself (i.e. in your own patient population). Then it provides you with extra information regarding the type of patients you have. You could stratify them on the basis of a risk score. And you could say, well, this category patients functions like this, and this category functions like that, and this so (cardiologist, teaching hospital, PCI facilities).

Table 3 Representative quotations (continued)

PGF dimension	Category	Representative quotes
III. Perceptions of health care providers *(content)*	Unintended and intended benefits and risks	*Well, because every treatment brings morbidity and mortality. Every pill, every PCI, you name it. Everything gives morbidity and mortality. And that only balances out, if the normal prognosis has a higher morbidity and mortality. Than you are allowed to administer that certain treatment. Otherwise you damage everybody with that treatment. Well, if you know this, and you have a risk model for it, than you should really use it. Because otherwise it means that, if you would give everybody the maximum treatment, you would over-treat two thirds of people who you damage (…) (cardiologist, teaching hospital, PCI facilities).*
		Yes, I think that a disadvantage can be that you overestimate people in terms of mortality risk and that you might, unnecessarily, earlier catheterize them or treat them invasively. And that you incorrectly consider people as unstable angina pectoris, while the diagnosis was different, but due to the high GRACE score you choose that (i.e. invasive) path, while otherwise you might have thought harder about an alternative diagnoses. However, it is difficult to say if that actually is the case, it might (medical resident, teaching hospital).
	Impact on treatment policies	*Fast administration of medication, fast and clear policies. That enhances the patient flow on the emergency department, and that is of course where I do it for. Because my emergency department is for fast diagnostics and rapid treatment, but also for quickly deciding on the correct location of care: to an intervention center, or upstairs (e.g. coronary care unit of cardiology ward), or home. That is, what I want to have clear as soon as possible. And not that people are waiting here for hours (emergency physician, general hospital).*
		Yes, exactly. It is decisive for the antiplatelet therapy. And in addition we use the GRACE score for the moment of catheterization. So if someone has a high GRACE score, than he will be considered earlier for catheterization (medical resident, teaching hospital, PCI facilities).
		Ehm, no. The standard policy is that you work conform the guidelines. The GRACE actually adds not much to it (cardiologist, general hospital).
	Effects on process of care	*There are people who don't take it into account, who have no feeling with it at all, who think it is nonsense… (cardiologist, teaching hospital, PCI facilities)*
		It will also have to do with individuals. That one person has more belief in it, and that others experience it as a burden: something has to be done again. That people find it sometimes difficult, like they are not taking good care of their patients. While I think that's not the case. Only it is not verifiable without such a scoring system. Anyway, that differs per individual. I think when a person has little feeling with scoring systems or numbers, they are less willing to adopt it and register it. I think it depends in great extent on that. If you look at the differences, the periods of scoring here in the hospital, you see that it very much fluctuates. And to me it seems that it has partly to do with that (cardiologist, general hospital).
